# mRNA and microRNA expression profiles of radioresistant NCI-H520 non-small cell lung cancer cells

**DOI:** 10.3892/mmr.2015.3600

**Published:** 2015-04-08

**Authors:** WEI GUO, LI XIE, LONG ZHAO, YUEHUAN ZHAO

**Affiliations:** 1 Ultrasound Diagnosis Department, Shandong Cancer Hospital and Institute, Jinan, Shandong 250117, P.R. China; 2 Shandong Provincial Key Laboratory of Radiation Oncology, Shandong Cancer Hospital and Institute, Jinan, Shandong 250117, P.R. China

**Keywords:** microarray, radioresistance, mitogen-activated protein kinase, neurotrophin

## Abstract

To elucidate the mechanism of radioresistance in non-small cell lung cancer (NSCLC) cells and to identify key molecules conferring radioresistance, the radioresistant subclone NCI-H520/R, derived from the NCI-H520 NSCLC cell line, was established with eight rounds of sublethal irradiation. The radioresistant features were subsequently assessed using a clonogenic assay, analysis of apoptosis and an MTT assay, the gene expression levels were examined using an Agilent Whole Human Genome 4×44 k Oligo microarray and Agilent Human miRCURY™ LNA array, and confirmed by reverse transcription-quantitative polymerase chain reaction. Pathway analysis and Gene Ontology (GO) analysis were performed to determine the biological functions of the subset of differentially expressed genes. miRNA-mRNA correlation analysis between the expression levels of each miRNA and all its predicted target genes was performed to further understand the radioresistance in the NCI-H520 cells. Following eight rounds of sublethal irradiation, a total of 2,862 mRNAs were significantly differentially expressed in the NCI-H520/R cells, including 893 upregulated genes and 1,969 downregulated genes. A total of 162 upregulated miRNAs and 274 downregulated miRNAs were significantly deregulated in the NCI-H520/R cells. Multiple core regulatory processes and signaling pathways were identified as being of likely relevance to radioresistance in NCI-H520/R cells, including the mitogen-activated protein kinase signaling pathway and neurotrophin signaling pathway. The expression of genes associated with radioresistance reflects the complex biological processes involved in clinical cancer cell eradication and requires further investigation for future enhancement of therapy.

## Introduction

Lung cancer is the leading cause of cancer-associated mortality, with non-small cell lung cancer (NSCLC) accounting for ~80% of all cases (1). Accordingly, radiotherapy performs a critical role in the curative management of patients with inoperable NSCLC, however, therapeutic outcomes of radiotherapy are often not entirely satisfactory, and radioresistance is considered to be a main factor restricting the successful treatment of radiotherapy (2).

Advances in bioinformatics and high-throughput technologies, including microarray analysis are improving current understanding of the molecular mechanisms underlying biological processes (3). MicroRNAs (miRNAs) are a class of endogenous, nonprotein-coding, 18–24 nt single-stranded RNAs. These derive from a stem-loop precursor to regulate gene expression by binding primarily to the 3′-untranslated region of specific target mRNAs, resulting in the disruption of mRNA stability and/or translation (4–7). Due to their post-transcriptional regulatory effects, miRNAs act to ‘fine-tune’ the levels of proteins involved in numerous biological processes, including embryogenesis, organogenesis, tissue homeostasis, immune system function and cell cycle control (4–6). Overexpression of miRNAs, including mir-155, in cancer implies their possible function as oncogenes through the negative regulation of tumor suppressor genes and/or genes, which inhibit cell differentiation or apoptosis (8,9). However, certain miRNAs, including let-7d and mir-127 are underexpressed in cancer and may function as tumor suppressors by inhibiting oncogenes and/or genes controlling cell differentiation or apoptosis (9). The expression levels of mRNAs and microRNAs have also been associated with therapeutic sensitivity and resistance (10,11).

To investigate the associates among the expression levels of the various mRNAs and microRNAs, as well as their correlations with radioresistance, the NCI-H520/R NSCLC radioresistant subline was established by fractionated irradiation. To expand and improve the mRNA and microRNA expression data, the NCI-H520 cells and radioresistant NCI-H520/R cells were profiled using an Agilent Whole Human Genome 4×44 k Oligo microarray, for mRNA, and an Agilent Human miRCURY™ LNA array, for miRNA, which interrogated ~41,000 genes and 1,891 microRNAs respectively.

## Materials and methods

### Cell lines and cell culture

The NCI-H520 human NSCLC cell line was maintained in Dulbecco’s modified Eagle’s medium (DMEM; Gibco Life Technologies, Grand Island, NY, USA), containing 10% heat-inactivated fetal bovine serum (Gibco Life Technologies, Grand Island, NY, USA), 100 U/ml penicillin (Sigma-Aldrich, Shanghai, China), 100 U/ml streptomycin (Sigma-Aldrich) and 2 mM L-glutamine, at 37°C in a humidified atmosphere of 5% CO_2_. The cells were passaged every 2–3 days to maintain exponential growth.

### Establishment of the radioresistant cell line

The method for establishing a radioresistant cell line by fractionated irradiation has been described previously (12). Briefly, the cells were first grown to ~50% confluence in 25 cm^2^ culture flasks. The cells were treated with 10 Gy X-ray irradiation using an X-ray generator (X-Ray 225; Precision X-Ray, North Branford, CT, USA), and then returned to the incubator at 37°C in a humidified atmosphere of 5% CO_2_ for 48 h to 72 h. When the cells reached ~90% confluence, the irradiation was repeated. The fractionated irradiation steps were continued until a total dose of 80 Gy was reached, at which point, the radioresistant cell sublines were established. The parental NCI-H520 cells were subjected to the same trypsinisation using 0.25% trypsin (Gibco Life Technologies) for 5 min at 37°C, replating and culture conditions, however they did not receive irradiation. For all the subsequent assays on the irradiated cells, there was an interval of at least 2 weeks between the final 10 Gy of irradiation and the start of the experiment.

### Assay for radiosensitivity

The survival of the cells following X-ray irradiation was measured using a clonogenic assay. The cells were plated in 60 mm tissue culture dishes, and were irradiated at different doses of 0, 4, 8, 10 and 12 Gy. These cells were incubated at 37°C for 10–14 days, with three plates included for each radiation dose. Following fixation of the cells with formalin and staining with 0.1% crystal violet (Sigma-Aldrich), colonies of cells consisting of ≥50 cells were counted under a light microscope (IX71; Olympus, Tokyo, Japan), and the surviving fraction was determined. All the survival curves were constructed from at least three independent experiments.

### Detection of apoptotic cells

The levels of apoptosis were determined using an Annexin V-fluorescein isothiocyanate (FITC) Apoptosis Detection kit (BD Biosciences, San Diego, USA). The cells were trypsinized using 0.25% trypsin and washed twice with cold phosphate-buffered saline (PBS, Beyotime Institue of Biotechnology, Beijing, China), followed by resuspension in 1X binding buffer. Annexin V (5 *μ*l) and propidium iodide (PI; 5 *μ*l) were then added and vortexed (Vortex-Genie 1; Scientific Industries, Inc., Bohemia, NY, USA). The cells were then incubated in the dark for 15 min at room temperature, following which, 400 *μ*l 1X binding buffer was added and the samples were evaluated by flow cytometry/fluorescence-activated cell sorting (FACS) using a FACSCalibur (BD Biosciences).

### MTT cell proliferation assay

To determine the cell proliferation, 3,000 cells/well were seeded into 96-well plates (Corning Incorporated; Corning, NY, USA) at an appreciated concentration and were grown for 0, 2, 4 and 6 days at 37°C with 5% CO_2_. The medium was subsequently removed and 10 *μ*l MTT solution (5 mg/ml in PBS; Sigma-Aldrich) and 100 *μ*l DMEM were added to each well for 4 h at 37°C. Following incubation, the formazan crystals were solubilized using 100 *μ*l 10% sodium dodecyl sulfate (SDS) in 0.01 M HCl for 24 h at 37°C and 5% CO_2_. The absorbance of the cells at 570 nm, relative to a reference wavelength of 630 nm, was then determined using a microplate reader (Bio-rad 680; Bio-Rad Laboratories, Inc., Hercules, CA, USA). Each experiment was performed in triplicate and was repeated at least three times.

### Microarray analysis, involving labeling, hybridization and scanning

The total RNA (1 *μ*g) from each sample was amplified and transcribed into fluorescent cRNA by Quick Amp labeling, according to the manufacturer’s instructions (Version 5.7; Agilent Technologies, Inc., Santa Clara, CA, USA). The labelled samples were then hybridized and subjected to the whole genome oligo array (4×44 K) and, following washing with PBS, the arrays were scanned using an Agilent Scanner G2505B; Agilent Technologies, Inc.).

The total RNA was harvested using TRIzol reagent (Invitrogen Life Technologies, Carslbad, CA, USA) and an miRNeasy Mini kit (Qiagen, Valencia, CA, USA), according to manufacturer’s instructions. The quantity of RNA was measured using a NanoDrop 1000 (Thermo Fisher Scientific Inc., Wilmington, DE, USA) and the samples were labeled, using a miRCURY™ Hy3™/Hy5™ Power labeling kit, and hybridized using the miRCURY™ LNA array (version 14.0). Following washing with PBS, the slides (Beyotime Institute of Biotechnology) were scanned using an Axon GenePix 4000B microarray scanner.

### Microarray data analysis

The array images acquired were then analyzed using Agilent Feature Extraction software (version 10.5.1.1; Agilent Technologies, Inc.). The median values of the raw data were normalized and subsequent data processing was performed using the GeneSpringGX v11.0 software package (Agilent Technologies, Inc.). Following median normalization of the raw data, the genes flagged in more than two samples were selected for further data analysis. The differentially expressed genes were identified through fold-change filtering. Fold change ≥2 or fold change ≤0.5 was considered as significantly differential expression. Pathway analysis and Gene Ontology (GO) analysis were performed to reveal the biological functions of this subset of differentially expressed genes.

The scanned images were then imported into GenePix Pro 6.0 software (Axon) for grid alignment and data extraction. The average number of replicated miRNAs was calculated and the miRNAs exhibiting intensities >50 in all samples were selected to calculate the normalization factor. The data on the expression were normalized using the median normalization, following which, differentially expressed miRNAs were identified through fold-change filtering.

### Reverse transcription-quantitative polymerase chain reaction (RT-qPCR)

To determine the expression levels of mRNA, RT-qPCR was performed, using GAPDH cDNA as an internal control. The SuperScript III Platinum SYBR Green One-Step qRT-PCR kit (Invitrogen Life Technologies) was used to quantify the levels of the selected differentially expressed genes, which were identified in the previous microarray analysis. In brief, three quantities of 0.2 *μ*g total extracted RNA were mixed with SuperScript III RT/Platinum, 2X SYBR Green reaction mix and gene specific forward and reverse primers (Invitrogen Life Technologies). The primer sequences were as follows: IL6, forward 5′-AAGAGTAACATGTGTGAAAGC-3′ and reverse 5′-CTACTCTCAAATCTGTTCTGG-3′ (188 bp); CLDN1, forward 5′-CACCCTTGGCATGAAGTGTA -3′ and reverse 5′-AGCCAATGAAGAGAGCCTGA-3′ (216 bp); HGF, forward 5′-GCTGACAATACTATGAATGAC-3′ and reverse 5′-TCGTGAGGATACTGAGAAT-3′ (143 bp); IGFBP2, forward 5′-GCCCCCTGGAACATCTCTACT-3′ and reverse 5′-TCCGTTCAGAGACATCTTGCA-3′ (92 bp); TP53, forward 5′-ACCCAGGTCCAGATGAAG-3′ and reverse 5′-GCAAGAAGCCCAGACG-3′ (174 bp); BAX, forward 5′-CTGAGCGAGTGTCTCAAGCG-3′ and reverse 5′-CCCCAGTTGAAGTTGCCGTC-3′ (149 bp) and GAPDH, forward 5′-TGCACCACCAACTGCTTAG-3′ and reverse 5′-AGTAGAGGCAGGGATGATGTTC-3′ (180 bp). Amplification was performed using an ABI Prism 7000 (ABI, Applied Biosystems) sequence analyzer using the following conditions: 30 sec at 95°C, 45 cycles of 10 sec 95°C; 10 sec at 62°C; 10 sec at 72°C. The relative expression levels of each gene was calculated by comparing the threshold cycle (Ct) value of samples with that of the GAPDH reference cDNA. All the data were normalized to GAPDH. The following formulae wrtr used to calculate the relative expression levels: ∆Ct = (mean Ct value of gene) − (mean Ct value of GAPDH), and ∆∆Ct = ∆Ct(selected cells) − ∆Ct(parental cells). The relative gene expression in a particular sample was then determined by calculating 2−^∆∆Ct^.

### Statistical analysis

Statistical significance was evaluated with data from at least three independent experiments. GraphPad Prism 6.02 (GraphPad Software, San Diego, CA, USA) was used for data analysis. The statistical analyses were performed using a t-test. The enrichment P-value of the Pathway ID was determined using Fisher’s exact test. Data are expressed as the mean ± standard error of the mean. P< 0.05 was considered to indicate a statistically significant difference.

## Results

### Establishment of the irradiation-resistant cell subline

**Figure 1:**
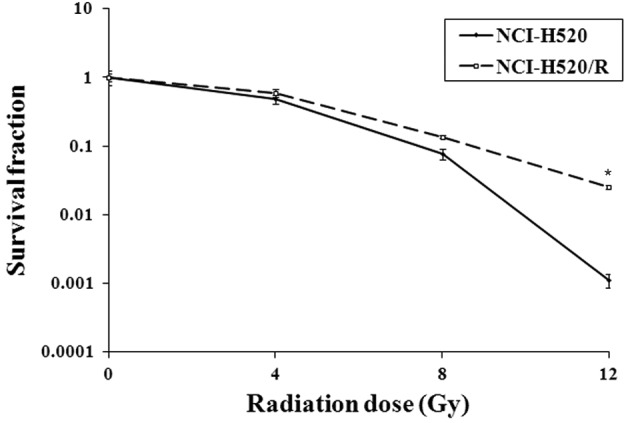
Survival curves for NCI-H520 and radioresistant NCI-H520/R cells. The clonogenic assay was described in Materials and methods. There was a significant difference in survival fraction between parent and radioresistant cells (^*^P<0.05). NCI-H520/R, radioresistant subclone.

The NCI-H520 cells were treated with fractionated irradiation, at a sublethal dose, resulting in survival of cells. The populations of cells, which survived the fractionated irradiation were analyzed for their radiosensitivity using a clonogenic assay to determine the rates of survival following irradiation at doses between 0 and 12 Gy. Fig. 1 shows the survival curves of the parental and radioresistant cells. There was a significant increase in radioresistance in the NCI-H520/R subline compared with the parental cells. Therefore, the NCI-H520/R subline was considered to be radioresistant. The radioresistant phenotype of these cells was maintained for at least 2 months following cessation of the fractionated irradiation (data not shown).

### Irradiation-induced apoptosis in the radioresistant NCI-H520/R cells

**Figure 2:**
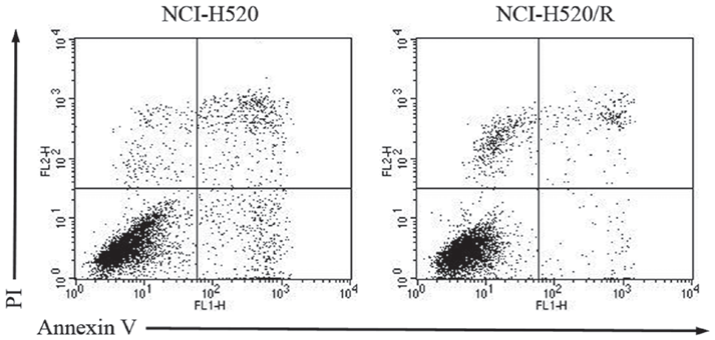
Irradiation-induced apoptosis in NCI-H520 and NCI-H520/R cells. The cells were seeded and incubated for 48 h following treatment with 10 Gy irradiation. Annexin V-fluorescein isothiocyanate and PI staining was performed, followed by fluorescence-activated cell sorting analysis. The percentage of early apoptotic cells (annexin V+, PI−) was calculated. Similar results were obtained in three independent experiments. PI, propidium iodide.

The present study subsequently determined the induction of apoptotis by 10 Gy irradiation using annexin V-FITC staining. The NCI-H520/R and parental NCI-H520 cells received 10 Gy irradiation, following which, the cells were collected for annexin V FACS analysis. As shown in Fig. 2, the rate of apoptosis in the NCI-H520/R and NCI-H520 cells were 2.84±0.58 and 8.25±1.01%, respectively, 48 h after 10 Gy irradiation treatment (P<0.05). Therefore, the acquirement of radioresistance was reflected in the reduced apoptotic rate.

### Cell proliferation in vitro

**Figure 3:**
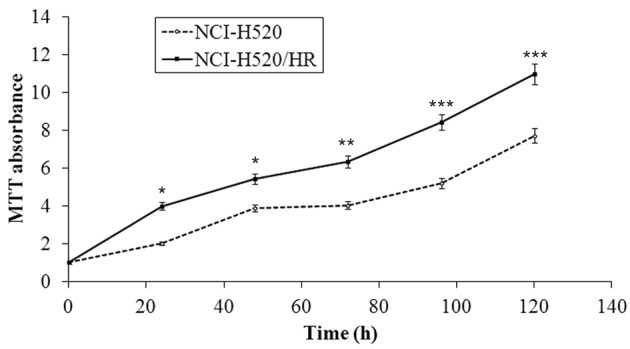
Cell proliferation assays of NCI-H520 and NCI-H520/R cells. The cells were cultured in 96-well plates for 0, 24, 48, 72, 96, and 120 h. The proliferation of the cells was determined using an MTT assay. The data are expressed as the mean ± standard deviation of three experiments. Statistically significant differences were obtained (^*^P<0.05;^**^P<0.01; ^***^P<0.001; n=6). NCI-H520/R, radioresistant subclone.

To assess differences in the proliferation of NCI-H520 and NCI-H520/R cells, the viability of the cells was determined using an MTT assay. Aliquots of 2×10^3^/well NCI-H520 and NCI-H520/R cells were cultured in 96-well plates for 0, 24, 48, 72, 96 and 120 h. The absorbance values of the MTT product were then detected. As shown in Fig. 3, there was a significant difference between the NCI-H520 and NCI-H520/R cells in cell growth following three repetitive treatments (P<0.05). Each point in Fig. 3 represents the mean ± standard deviation of the three experiments. The radioresistant cells exhibited increased cell growth compared with the parental cells.

### mRNA and miRNA profiling

The present study performed global expression analysis of 41,000 genes and 1,891 microRNAs using the Agilent Whole Human Genome 4×44k Oligo microarray and Agilent Human miRCURY™ LNA array, respectively. Compared with the parental cells, a total of 2,862 mRNAs were significantly differentially expressed, including 893 upregulated genes (fold-change ≥2) and 1,969 downregulated genes (fold-change ≤0.5). A total of 162 upregulated miRNAs (fold change ≥2) and 274 downregulated miRNAs (fold change ≤0.5) were significantly deregulated in the NCI-H520/R cells.

**Figure 4:**
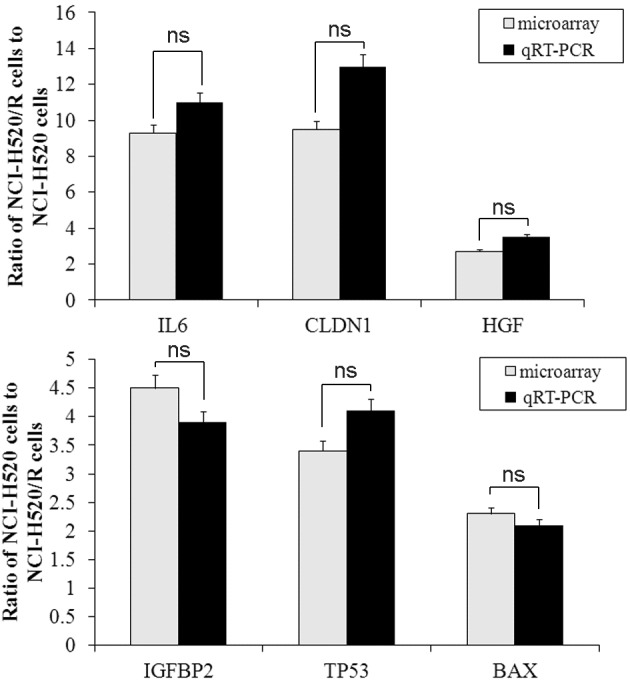
Confirmation of differentially expressed genes by RT-qPCR. For each gene, the white bar indicates the expression ratio of cancer cells, determined in the microarray, while the black bar indicates the expression ratio, determined by RT-qPCR. All qRT-PCR data were consistent with the microarray data. NCI-H520/R, radioresistant subclone; RT-qPCR, reverse transcription-quantitative polymerase chain reaction IL, interleukin; CLDN1, claudin 1; HGF, hepatocyte growth factor; IGFBP, insulin-like growth factor binding protein, TP53, tumor protein 53 BAX, B-cell-associated X protein; ns, not significant.

Among the deregulated genes identified by microarray analysis, six known genes were selected for validation by RT-qPCR. These genes included three upregulated genes: *IL6*, *CLDN1* (claudin 1) and hepatocyte growth factor (*HGF*), and three downregulated genes: Insulin-like growth factor binding protein (*IGFBP2*), tumor protein 53 (TP53) and B-cell-associated X protein (*BAX*). Notably, the RT-qPCR demonstrated significant correlation with the microarray analysis (Fig. 4). Specifically, IL6, CLDN1 and HGF were upregulated 9.3–11- and 9.5–13- and 2.7–3.5-fold, respectively in the NCI-H520/R cells compared with the NCI-H520 cells. The downregulated genes included IGFBP2, TP53 and BAX, the expression levels of which were decreased 4.5–6-, 3.4–4.6- and 2.3–2.5-fold, respectively in the NCI-H520/R cells compared with the NCI-H520 cells. Therefore, the results of the micro-array and RT-qPCR analyses were generally highly correlated, supporting the reliability and rationale of the techniques used in the present study.

### Pathway enrichment of genes significantly deregulated in the NCI-H520/R cells

All the genes, which were deregulated in then NCI-H520/R cells were used separately as inputs for the Database for Annotation, Visualization and Integrated Discovery (DAVID, http://david.abcc.ncifcrf.gov/) bioinformatics resource to identify the significantly deregulated pathways (13). The resulting lists of KEGG pathways, which were significantly enriched (P<0.05) in the deregulated genes are listed in Table I. The nucleotide-binding oligomerization domain (NOD)-like receptor signaling pathway was affected significantly, and other disrupted pathways included the mitogen-activated protein kinase (MAPK), B-cell receptor, Janus kinase-signal transducers and activators of transcription (JAK-STAT), Neurotrophin, Insulin, Hedgehog and ErbB signaling pathways, and the Apoptosis pathway.

### GO enrichment analysis of the genes differentially expressed in the NCI-H520/R cells

Gene sets, which exhibit identical patterns of deregulated expression in multiple genes, and exhibit deregulation of the expression of cancer-specific genes are considered to be functionally important in tumorigenic processes (14). Thus, the present study examined the genes, which exhibited deregulation patterns in the NCI-H520/R cells, to identify the significantly deregulated GO biological process terms. Table II shows the 10 most significant GO terms in more detail. For the genes, which exhibited deregulation patterns, the majority of the markedly enriched GO biological terms were associated with the developmental process. Other important biological processes, which were significantly enriched in the deregulated genes included the intracellular signaling pathway, cell death, apoptosis, cell communication, catabolic process, cell differentiation, cell motility and migration process pathways.

### Correlation analysis between the expression levels of differentially expressed miRNAs and their potential target genes

miRNAs are considered a useful tool to regulate the levels of gene expression via the RNA interference pathway. Therefore, the present study performed miRNA-mRNA correlation analysis between the expression levels of each miRNA and its predicted target genes, including the differentially expressed genes obtained in the mRNA microarray analysis, to further understand the radioresistance in NCI-H520 cells. Significant correlations (P<0.05) were found between 981 miRNA-mRNA pairs. Of these, 100 mRNAs were negatively regulated by miRNAs with decreased expression, thereby increasing the corresponding mRNA expression, and 881 mRNAs were regulated by miRNAs with increased expression, thereby decreasing the corresponding mRNA expression.

**Figure 5:**
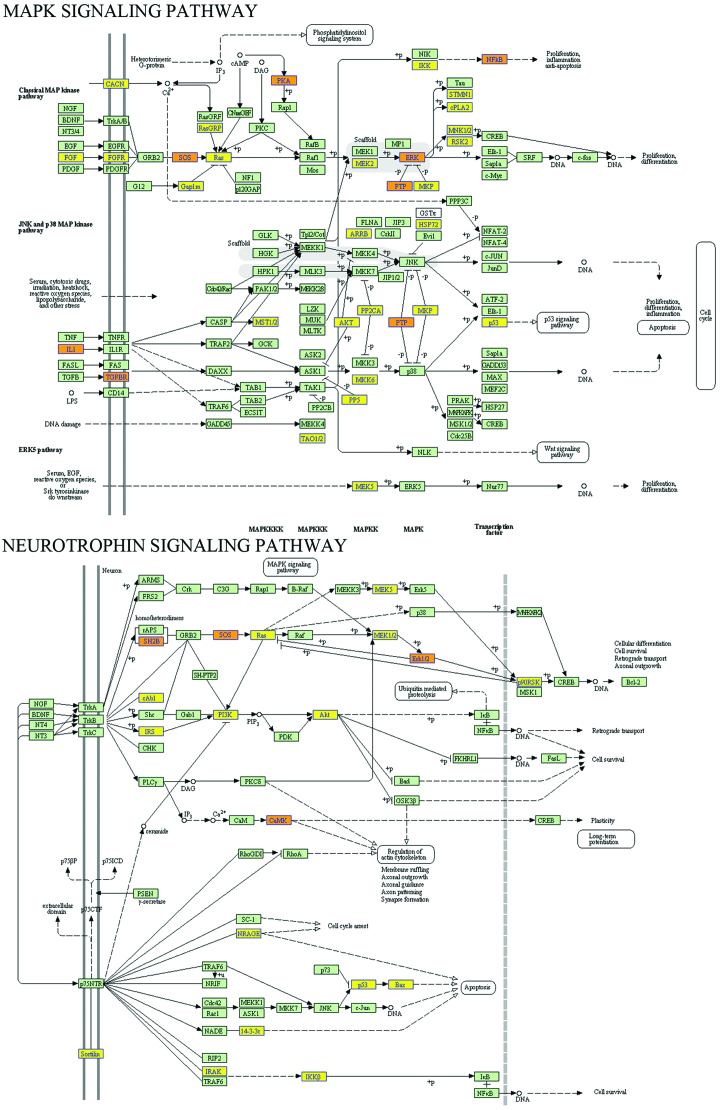
MAPK signaling pathway and neurotrophin signaling pathway maps. The yellow nodes are associated with downregulated genes and the orange nodes are associated with upregulated or only whole dataset genes. The green nodes indicate no significant change in expression.. MAPK, mitogen-activated protein kinase.

The GO biological processes enriched with these significantly correlated target genes were subsequently determined. Multiple core regulatory processes and signaling pathways were identified as likely to be of relevance to radioresistance, including the MAPK signaling pathway and neurotrophin signaling pathway (Fig. 5).

## Discussion

The present study provided an integrated overview of the radioresistance in NCI-H520/R cancer cells, based on the whole-genome expression profiles of mRNA and miRNA. The integrative analysis of the expression profiles of mRNA and miRNA assisted in identifying deregulated biological processes or pathways, which may be under the regulation of miRNAs and, as a result, may serve as ideal therapeutic targets.

The genes, which were identified in the present study provide new insight into the reasons underlying the observed tumorigenic phenotypes of NCI-H520/R cells, including decreased radiosensitivity, resistance to cell death and enhanced cell proliferation. In addition, the number of genes differentially expressed in the NCI-H520/R cells was found to associate radiosensitivity with a number of signaling pathways, which have been previously implicated in the tumorigenic process and clinical cancer settings (15). Notably, this technique identified of novel pathways by which radiosensitivity may occur.

Ionizing radiation, as with several other cellular stresses, can activate or downregulate multiple signaling pathways, leading to either increased cell death or increased cell proliferation. The MAPK cascade is a highly conserved pathway, which is involved in various cellular functions, including cell proliferation, differentiation and migration. At least four distinctly regulated groups of MAPKs are expressed in mammals: Extracellular signal-regulated kinases (ERK)-1/2, Jun amino-terminal kinase (JNKs)1/2/3, p38 proteins (p38α/β/γ/δ) and ERK5 (16,17). A number of signal transduction pathways, which are stimulated by ionizing radiation are mediated by the MAPK superfamily. Low-dose radiation selectively induces p38-MAPK mediated cell migration in tumour cells (18). MAPK signaling is considered to have a potential effect on tumor cell radiosensitivity, due to the association of their activity with the response to radiation-induced DNA damage (19). Small molecule inhibitors of MAPK kinase (MEK) have been assessed previously for their radiosensitizing potential in *in vitro* and *in vivo* investigations, and enable the involvement of the ERK pathway in the radiation response to be assessed. As singlie agents, MEK inhibitors have been observed to exhibit radiosensitizing properties in a broad spectrum of human tumor types (13,20).

The neurotrophin signaling pathway may function in radioresisitance, as several of the genes identified in the microarray analysis of the present study are regulated by neurotrophins, including CaMK, KRAS and TP53 (21,22). Neurotrophins are a family of trophic factors, which are involved in differentiation and survival of neural cells (23,24). This family consists of nerve growth factor (NGF), brain derived neurotrophic factor (BDNF), neurotrophin 3 (NT-3), and neurotrophin 4 (NT-4). Neurotrophins functions through binding to the Trk tyrosine kinase receptors or the p75 neurotrophin receptor (p75NTR). Regulation of neurotrophin/Trk signaling occurs though a connection of several intracellular signaling cascades, including the MAPK pathway, PI-3 kinase pathway and PLC pathway, which transmit positive signals, including enhanced survival and growth (25,26). However, p75NTR transmits positive and negative signals, which are important for neural development, learning and memory. The results of these studies and the present study indicate that the z the radioresistance of NCI-H520 cells.

The pathways highlighted in the present study indicated potential mechanisms though which radioresistance may occur. The number of genes identified in the present study suggested the MAPK signaling pathway and neurotrophin signaling pathway as essential pathways within the cell, regulating several functions though a number of genes. Future investigations are required to examine the associations between the MAPK signaling pathway, neurotrophin signaling pathway, their differentially expressed genes, and key signaling molecules. Following which, the effect of these associations on the radioresistance of cells requires elucidation.

In the present study, a comparative analysis of the whole genomic expression profiles of mRNA and miRNA was performed in radioresistant NCI-H520/R cancer cells. The findings demonstrated a number of common changes in a small set of conserved biological processes and pathways in human radioresistant NSCLC cancer, and revealed the effects of certain commonly deregulated genes and miRNAs on the radioresistance of the tumor cells. In addition, the results of the present study identified a number of pathways, processes and individual markers, which exhibited changes in expression associated with radioresistance. Taken together, the findings of the present integrated comparative study demosntrated the system-level molecular phenotypes of NCI-H520/R cancer cells and simultaneously examined variations in the expression levels of miRNA. These findings offer valuable information for future investigations on NSCLC cancer, and the genes, pathways and processes identified may provide diagnostic or therapeutic purposes.

## Figures and Tables

**Table I:** Pathways significantly enriched with differentially expressed genes in the NCI-H520/R cells.

Pathway ID	Definition	Fisher’s P-value
hsa04621	NOD-like receptor signaling pathway	0.0023104
hsa04010	MAPK signaling pathway	0.0134484
hsa04662	B cell receptor signaling pathway	0.0146623
hsa04630	JAK-STAT signaling pathway	0.0175341
hsa04722	Neurotrophin signaling pathway	0.0178688
hsa04910	Insulin signaling pathway	0.0241512
hsa04340	Hedgehog signaling pathway	0.0379188
hsa04012	ErbB signaling pathway	0.0493879
hsa04210	Apoptosis	0.0493879

Significantly enriched pathways (P<0.05) are listed, following manual removal of human disease pathways. NOD, nucleotide-binding oligomerization domain; MAPK, mitogen-activated protein kinas; JAK, Janus kinase; STAT, signal transducers and activators of transcription.

**Table II:** Deregulated genes in GO analysis.

GO ID	Term	P-value	Gene
GO:0050793	Regulation of developmental process	9.6E-09	SOX2, VEGFC, CD24, TP53, DLC1, CDC42EP2, PDPN, CDC42EP1, AKAP2, FGD4, GAS2, ITGB2, PALM, FBLIM1, RHOU, ARAP3, GAS7, FGD3, TAOK2, AGRN, E4F1, CST3, PLA2G10, PPARG, ABCG1, HDAC5, NKX2-5, CENPF, SPINK5, KLK3, FOXO4, PLG, THBS1, LIF, GLI2, PBX1, ADRB2, ENPP1, BMP2K, ANKH, BMPR1B, ACVR2A, ECM1, AHSG, SOX9, NTN1, ALOX15B, NEFL, KLK8, PTHLH, IL7R, SPN, DEAF1, IL1B, TNFRSF11B, CD83, INPP5D, CD27, TCFL5, KLK6, FST, IAPP, WNT4, MAPK1, CLU, SOCS3, TAZ, FNDC3B, AQP3, NOTCH1, MUM1, IL4R, RARA, MAFB, ETS1, L3MBTL, TESC, ACIN1, MBNL3, IGFBP3, ATN1, FOXA2, LMX1A, MIB1, ISL2, NRP1, IL6, HGF, CCL5, NAB2, RNH1, F3, DDAH1, IL1A, RHOB, ANGPTL4, LUC7L, SOX15, INSR, LAMA3, LAMA3, LAMA5, BAX, BCL2L11, STAT6, TNFSF13, CD40, ULK1, FEZ1, CHRNB2, SEMA3A, NRP1, EFNA1, MAG, ARHGEF1, AMIGO1, DBN1, TGFBR2, NRG1, MORF4L2, CXADR, RBP4, HGF, MESP1
GO:0023034	Intracellular signaling pathway	0.00000012	CD24, SIGIRR, AGPAT2, TLR6, HSPD1, CD40, APC2, FZD10, CSNK1G2, AES, DVL1, CELSR2, HBP1, INVS, DACT1, PIAS4, KLHL12, FBXW4, TCF7, WNT4, WNT6, WNT7B, WNT11, WNT10A, ZFYVE28, TP53, ERN1, EPHB3, ERBB4, MUSK, NRTN, ROR1, MAL, ADRB2, PILRB, PDGFC, GRB7, AREG, CAMLG, PSEN2, ARRB2, CCL2, TGFBR2, GDF15, EID2, FKBP1A, PTPRE, TRIO, GRID2, F3, CFD, NRG1, MESP1, MIB1, NCSTN, NOTCH1, FOXF1, HIPK2, IFT52, VAV3, ITGA2, ITGAE, ITGB2, LAMA5, CEACAM1, CD47, TRPV4, BAIAP2, AKT2, INSR, FOXO4, PHIP, PIK3R3, IRS2, FGF12, FGFR3, PTCH1, F3, SNCA, SMAD6, CEBPA, KLF6, CSF2RB, EDN2, IL1A, IL1B, KRAS, IRAK4, DUOX1, STAT4, STAT6, GLI2, PPM1A, SOX4, ZEB2, DKK3, TAX1BP3, LRP1, WWOX, CHD8, SOX17, MED12, FST, BMPR1B, HOXA13, PCSK6, RGMA, ACVR2A, CDKN1C, GIPC1, THBS1, CHST11, VCP, MAPK1, TRIB1, PYDC1, CLN3, AGRN, PICK1, IGFBP2, AFAP1L2, SOX2, GAS1, AHSG, GRB14, IRS1, ENPP1, SOCS3, MYO1C, ZFAND5, SGPL1, VEGFC, NRP1, SORT1, HGF, EFNA1, EPHA1, CTF1, LIF, CACNB3, ELF1, ULK1, PROP1, RARG, HSP90AB1, IL6, HGF
GO:0016265	Death	0.00000074	IL6, HGF, BCL2L11, BAX, CLU, BBC3, TP53, CADM1, CD24, SEMA3A, LRP1, VAV3, IL24, AKAP13, FGD4, TRIM69, DNASE2, ECE1, ERN1, EYA2, UNC5B, PHLDA1, ACIN1, ARHGEF9, MCF2L, NCSTN, DAPK2, NGEF, GAS2, TIAM2, TNFRSF21, PDCD4, CECR2, HIPK2, UBQLN1, HRAS, IAPP, IL1A, ITGB2, USP17, NAIP, TNFRSF11B, PDCD2, WWOX, PLG, CYCS, PPP2R2B, DRAM1, LRDD, EAF2, PREX1, PTPN6, PTPRH, ELMO2, PERP, AKTIP, SGK1, LGALS7B, HGF, THBS1, TRIO, YWHAE, CXCR4, FASTKD1, ELMO3, HINT2, GRIP1, SGPL1, FGD3, RABEP1, STK17B, STK17A, TAOK2, CD27, NTN1, BRE, ARHGEF11, EEF1A2, F3, ATF1, HGF, HMGB1, HSPA1A, AMIGO2, IKBKB, IL1B, SERPINB2, CCL2, SNCA, ARC, SOCS3, DLC1, UNC13B, EIF5A, NLRP1, ALOX15B, PCSK9, MAL, NRP1, PPARG, MAPK1, BIK, BOK, TGM2, CASP1, TNFSF10, F2R, PSAP, HSPD1, VCP, RHOB, KLK8, CLN3, TTBK2, CTSD, DPP6, ATN1, L1CAM, OLR1, PSEN2, CABC1, SMPD1, SPG7, FA2H, FIG4, GAS1, C5, SORT1, SPN, CD5, MAGED1, ABL1, PRODH, DLC1, CASP9, NKX2-5, CST3, PSME3, CST6, CTSB, CARD8, CARD10, MNT, SOX9, TRAF1, ACTN4, BMF, SHARPIN, HDAC6, ADRB2, HOXA13, IGFBP3, INPP5D, MMP9, NOTCH1, IP6K2, ADAMTSL4, MAP2K6, PTGS2, KIAA1967, RARG, RXRA, SOX4, STK4, FOSL1, DHRS2, SMAD6, CHST11, ANGPTL4, PIM1, FAIM, PROP1, CHD8, PSMC5, SOX2, UNQ1887, GRID2, KRAS, NEFL, PIGT, AFG3L2
GO:0006915	Apoptosis	0.000001	IL6, HGF, BCL2L11, BAX, CLU, BBC3, TP53, CADM1, CD24, CECR2, CYCS, EEF1A2, F3, ATF1, HGF, HMGB1, HSPA1A, AMIGO2, IKBKB, IL1A, IL1B, NAIP, SERPINB2, CCL2, SNCA, THBS1, ARC, SOCS3, CD27, DLC1, UNC13B, EIF5A, ERN1, PHLDA1, NLRP1, DAPK2, ALOX15B, PCSK9, MAL, NRP1, WWOX, PLG, PPARG, MAPK1, BIK, PERP, BOK, TGM2, CASP1, TNFSF10, STK17B, STK17A, F2R, PSAP, HSPD1, VCP, RHOB, VAV3, AKAP13, FGD4, ARHGEF9, MCF2L, NCSTN, NGEF, TIAM2, PSEN2, PREX1, SORT1, HGF, SPN, TRIO, YWHAE, FGD3, CD5, MAGED1, ARHGEF11, HIPK2, GRIP1, ABL1, PRODH, DLC1, CASP9, NKX2-5, PSME3, CST6, CTSB, CARD8, GAS1, CARD10, MNT, SOX9, TRAF1, ACTN4, BMF, SHARPIN, ACIN1, HDAC6, ADRB2, HOXA13, IGFBP3, INPP5D, MMP9, NOTCH1, IP6K2, ADAMTSL4, MAP2K6, PTGS2, KIAA1967, RARG, RXRA, SMPD1, SOX4, STK4, FOSL1, DHRS2, SMAD6, CHST11, ANGPTL4, PIM1, FAIM, PROP1, CHD8, SOX2, UNQ1887, GRID2, CLN3, HRAS, KRAS, NEFL, PIGT, SEMA3A, LRP1, IL24, TRIM69, DNASE2, ECE1, EYA2, UNC5B, GAS2, TNFRSF21, PDCD4, UBQLN1, IAPP, ITGB2, USP17, TNFRSF11B, PDCD2, PPP2R2B, DRAM1, LRDD, EAF2, PTPN6, PTPRH, ELMO2, AKTIP, SGK1, LGALS7B, CXCR4, FASTKD1, ELMO3, HINT2, SGPL1, RABEP1, TAOK2, NTN1, BRE
GO:0010646	Regulation of cell communication	0.000002	EFNA1, HGF, IL1B, INSR, MAP2K6, THBS1, C5, CXCR4, DUSP2, DUSP6, PCSK9, ECE1, SNCA, CD24, SIGIRR, AGPAT2, ARRB2, ADRB2, ZFYVE28, PSEN2, TLR6, CARD10, FGD4, ABP1, PKN1, PTCH1, PSAP, HDAC6, KIF26A, IGFBP3, INPP5D, RGS9BP, F3, SMAD6, CLN3, FST, EDN2, DVL1, PPM1A, SOX4, ZEB2, DKK3, TAX1BP3, LRP1, WWOX, CHD8, SOX17, MED12, HOXA13, PCSK6, RGMA, ACVR2A, CDKN1C, GIPC1, HIPK2, EID2, CHST11, KLK8, TRIB1, PTGS2, SLC7A1, IQSEC2, IQSEC1, GK, PFKFB2, RBP4, TACR1, CHRNB2, HRAS, KRAS, ERBB4, ADAP2, ARAP3, TBC1D7, TBC1D8B, GRTP1, TBCK, SGSM2, RABGAP1L, RAP1GAP, PAFAH1B1, FKBP1A, NCOA3, FARP1, VAV3, AKAP13, PLEKHG4B, ARHGEF9, MCF2L, NGEF, TIAM2, ITSN2, ARHGEF10L, PREX1, HGF, TRIO, FGD3, ARHGEF1, ARHGEF10, ARHGEF11, DLC1, WNT4, IL6, HGF, LIF, ARHGAP27, IKBKB, TRIM38, ECM1, F2R, ZDHHC13, PPP5C, TGM2, TRAF1, CASP1, TNFSF10, VAPA, CD40, CARD8, SOX2, PDCD4, IGFBP2, AFAP1L2, KLK5, KLK6, GAS1, AKT2, TAOK3, EDA2R, TAOK2, CD27, AHSG, GRB14, IRS1, ENPP1, PTPRE, SOCS3, KCNQ1, KCNMB4, PPOX, SNCG, FEZ1, P2RX3, CALB1, DBN1, ARC, BCAN, BHLHE40, SYNGR1, CSPG5, NLGN2, CCL2, L1CAM, P2RX5, MAL, ELF1, RASGRP2, RGL1, RGL3, RALGDS, RASA2, ULK1, ATF1, ITGA2, HSP90AB1, MESP1
GO:0044243	Multicellular organismal catabolic process	0.000012	CST3, MMP1, MMP2, MMP3, MMP7, MMP9, MMP10, MMP13, MMP19, PRSS2, KLK6, CEL
GO:0030154	Cell differentiation	0.000072	DAB2, NKX2-5, FOXF1, MYO1C, SOX17, TGFBR2, TNNC1, SGPL1, CUL7, CYP24A1, FHL2, TAZ, WWOX, PAFAH1B1, NOTCH1, PSEN2, SOX2, SOX9, ATF1, LAMA5, NRTN, OVOL2, ZEB2, MDGA1, GFRA3, PAF1, PEX5, YWHAE, DCLK1, NTN1, HAND1, HGF, VEGFC, HSPG2, CHST11, TFCP2L1, GLI2, FST, MLF1, TP53, SOX4, CD24, SPN, DEAF1, RBP4, SPANXB2, HSPA2, PPP2R2B, STRBP, BBS4, NEURL, BAIAP2, AFG3L2, PIP5K1C, PARD6B, SEMA3A, UNC5B, GAS1, L1CAM, LMX1A, PLA2G10, NRP1, FEZ1, CELSR3, AMIGO1, EYA2, ADAM12, TBX2, DVL1, AGRN, MUSK, F2R, C1S, ACAT2, PPARG, ABCG1, HDAC5, NEFL, CHST3, NRG1, NAB2, SORT1, NTN4, SPN, LIF, HOXC10, ISL2, FOXA2, ROR1, ULK1, CHRNB2, EPHB3, PROP1, PCSK9, MESP1, PRDM12, CEBPA, TPO, THPO, EFNA1, NGRN, PIGT, PTPRR, SMARCA1, GAS7, NAPA, KLF6, BLNK, CDSN, SCEL, IL7R, TRIM10, DNASE2, EPAS1, ALAS2, ACIN1, MMP9, PLDN, CBFA2T3, SPINK5, ELF3, ALOX15B, VCAN, RXRA, GPRIN1, GNAO1, IL6, HGF, EFHD1, BMPR1B, PPL, SPRR1A, SPRR1B, TGM1, SHARPIN, PTHLH, GNGT1, CDKN1C, SMARCD3, DHRS2, MUM1, RELB, CD83, SPN, MAL, PSAP, PICK1, ERBB4, NEURL2, ACTG1, INSR, HINFP, MBNL1, PLG, INPP5D, CD27, TCFL5, KLK6, IAPP, WNT4, MAPK1, CLU, SOCS3, ENPP1, FNDC3B, AQP3, IL4R, RARA, MAFB, ETS1, ACVR2A, L3MBTL, TESC, MBNL3, IGFBP3, ATN1, PBX1, MIB1, NRP1, CCL5, BAX, PCSK4, SOX15, CTF1, NEUROD2, PTX3, FIG4, KLK8, CXADR, DTNBP1, CELSR2, FEZ1, MSI2, HOXD9, TBCE, MAG, ARHGEF1, DBN1, FABP4, ADRB2, LAMA3, ALDH6A1, TBX18, HRAS, KRAS, FOXO4, MORF4L2, OBSL1, CYP1A1, AHRR, ITGA2, SH2B3, CENPF, CSPG5, EID2B, EID2, DPYSL2, DUSP6, ATOH7, FHL1, AZI1, CADM1, NGEF, TMEM176B, CATSPER3, RHOB, STMN1, MAL, MDK, MMP19, ZBTB7B, ANGPTL4, SPATA6, PAQR5, CAND1, UNC45A, NDRG2, EDA2R, DMRTA1, GMCL1, BMP5, TLL2, BHLHE41, KAZALD1
GO:0048870	Cell motility	0.00023	CATSPER3, ATF1, LAMA5, NRTN, OVOL2, ZEB2, MDGA1, GFRA3, PAFAH1B1, PAF1, PEX5, YWHAE, DCLK1, NTN1, CD24, IL6, HGF, CCL2, ITGA2, MIA3, EDN2, MESP1, VEGFC, HDAC6, TACR1, THBS1, C5, ZFAND5, SGPL1, PLAU, TRIB1, IGFBP3, VAV3, PSG2, CEACAM1, TNS1, NRP1, TAOK2, ULK1, DNAJA1, ATP1A4, NEURL, LAMA3, LAMA3, PARD6B, NEXN, PDPN, ERBB4, F2R, F3, INSR, IRS1, MAPK1, IRS2, DLC1, ALOX15B, RNF20, ARAP3, IL1B, ITGB2, SAA1, SDS, COL5A1, PLG, GIPC1, CCL5, MSN, MMP9, BBS4, ARRB2, HDAC5, ETS1
GO:0006954	Infammatory response	0.0003	IL1A, IL1B, SPN, F2R, CD24, TACR1, F3, KLK3, CCL5, THBS1, PPARG, IL6, HGF, OSMR, GPX2, AHSG, F8, SIGIRR, SAA1, CFD, FHL1, C5, CLU, CD55, C1RL, C1R, C1S, C4BPB, PTGS2, ADRB2, FABP4, ITGA2, TGM2, HDAC5, TLR6, PDPN, MAL, LTB4R, S1PR3, ELF3, ALOX5, NCR3, XCR1, CXCL1, CXCL2, BLNK, HRH1, IL1RAP, ITGB2, OLR1, PTAFR, PTX3, RXRA, S100A8, CCL2, CCL21, BMPR1B, CXCR4, AFAP1L2, CHST1, PLA2G4C, PLAA, CHST2, CD40
GO:0016477	Cell migration	0.00055	ATF1, LAMA5, NRTN, OVOL2, ZEB2, MDGA1, GFRA3, PAFAH1B1, PAF1, PEX5, YWHAE, DCLK1, NTN1, CD24, IL6, HGF, CCL2, ITGA2, MIA3, EDN2, MESP1, VEGFC, HDAC6, TACR1, THBS1, C5, ZFAND5, SGPL1, PLAU, TRIB1, IGFBP3, ULK1, LAMA3, LAMA3, PARD6B, NEXN, PDPN, ERBB4, F2R, F3, INSR, IRS1, MAPK1, IRS2, DLC1, ALOX15B, RNF20, ARAP3, IL1B, ITGB2, SAA1, SDS, COL5A1, PLG, GIPC1, CCL5, MSN, MMP9, ARRB2, HDAC5, VAV3, PSG2, CEACAM1, TNS1, NRP1, TAOK2

GO, Gene Ontology.
